# Conserved Candidate Antigens and Nanoparticles to Develop Vaccine against *Giardia intestinalis*

**DOI:** 10.3390/vaccines11010096

**Published:** 2022-12-31

**Authors:** Suthinee Sangkanu, Alok K. Paul, Julalak Chuprom, Watcharapong Mitsuwan, Rachasak Boonhok, Maria de Lourdes Pereira, Sonia Marlene Rodrigues Oliveira, Polrat Wilairatana, Mohammed Rahmatullah, Christophe Wiart, Muhammad Nawaz, Chea Sin, Sunil Kayesth, Veeranoot Nissapatorn

**Affiliations:** 1 School of Allied Health Sciences, Southeast Asia Water Team (SEA Water Team) and World Union for Herbal Drug Discovery (WUHeDD), Walailak University, Nakhon Si Thammarat 80160, Thailand; 2 School of Pharmacy and Pharmacology, University of Tasmania, Hobart, TAS 7001, Australia; 3 School of Languages and General Education (SOLGEN), Walailak University, Nakhon Si Thammarat 80160, Thailand; 4 Akkhraratchakumari Veterinary College, Walailak University, Nakhon Si Thammarat 80160, Thailand; 5 Department of Medical Technology, School of Allied Health Sciences, Walailak University, Research Excellence Center for Innovation and Health Products (RECIHP), Nakhon Si Thammarat 80160, Thailand; 6 CICECO-Aveiro Institute of Materials, University of Aveiro, 3810-193 Aveiro, Portugal; 7 Department of Medical Sciences, University of Aveiro, 3810-193 Aveiro, Portugal; 8 Hunter Medical Research Institute, New Lambton, NSW 2305, Australia; 9 Department of Clinical Tropical Medicine, Faculty of Tropical Medicine, Mahidol University, Bangkok 10400, Thailand; 10 Department of Biotechnology & Genetic Engineering, University of Development Alternative, Dhaka 1209, Bangladesh; 11 The Institute for Tropical Biology and Conservation, University Malaysia Sabah, Jalan UMS, Kota Kinabalu 88400, Malaysia; 12 Department of Nano-Medicine Research, Institute for Research and Medical Consultations (IRMC), Imam Abdulrahman Bin Faisal University, Dammam 31441, Saudi Arabia; 13 Faculty of Pharmacy, University of Puthisastra, Phnom Penh 12211, Cambodia; 14 Department of Zoology, Deshbandhu College, University of Delhi, New Delhi 110019, India

**Keywords:** *Giardia intestinalis*, giardiasis, *Giardia* antigens, nanoparticles, vaccine

## Abstract

*Giardia intestinalis* (*Giardia lambia*, *Giardia duodenalis*) infections in humans may be asymptomatic or symptomatic and associated with diarrhea (without blood), abdominal cramps, bloating, flatulence, and weight loss. The protozoan Giardia is the third most common cause of diarrhea and death in children under five, preceded only by rotavirus and by *Cryptosporidium parvum* and *C. hominis* infections. Antimicrobial drugs, particularly 5-nitroimidazole (5-NIs), are used to treat giardiasis in humans. Immunologically naive or immunocompromised host are more vulnerable to *Giardia* infection, whereas a degree of resistance to this protozoan is present in humans living in endemic areas. This suggests that vaccination may be a potential and appropriate means to control this parasitic disease outbreak and protect the human population. This review discusses *Giardia* antigens related to vaccine development. Additionally, based on the latest development of nanoparticle technology, a combination of methods for future research and development is proposed for the design of the next generation of powerful immunogens and an effective vaccine against *Giardia*.

## 1. Introduction

**Figure 1:**
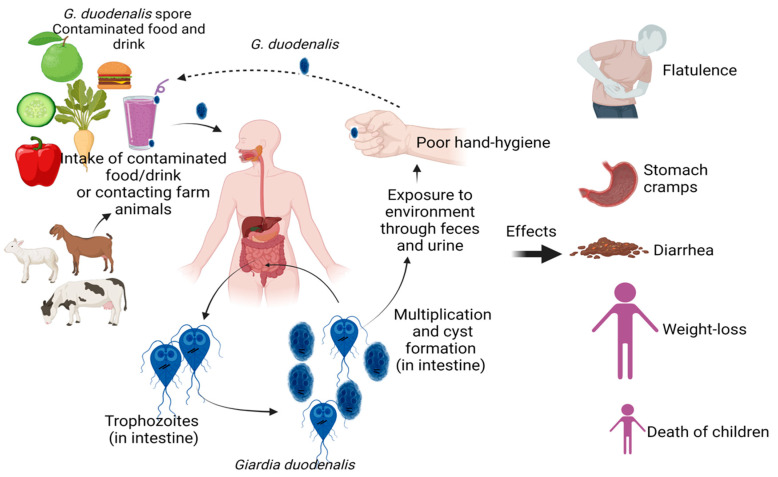
Effects of *Giardia intestinalis* infections in humans. The figure was made with www.biorender.com (accessed on 25 November 2022).

*Giardia intestinalis* (syn. *Giardia lamblia*, *Giardia duodenalis*) is a flagellated parasitic microorganism that causes giardiasis in humans, pets, livestock, and wildlife [1]. *G*. *intestinalis* has two morphological forms: trophozoite and cyst. The trophozoite has two anteriorly positioned symmetric nuclei and eight flagella that are organized into four bilaterally symmetrical pairs [2]. *Giardia*’s trophozoite is pear-shaped and is typically 12 to 20 µm long and 5 to 10 µm wide. This microorganism is transmitted via cysts, which are oval-shaped and smooth-walled. The thin-walled cyst is 8 to 12 µm long and 7 to 10 µm wide [3]. Cysts are moderately resistant to inactivation by disinfectants, for example, chlorine [4]. Infection occurs when a host swallows *Giardia*’s cysts from contaminated water or food [5]. They cause giardiasis, which is characterized by gastrointestinal disorders, including diarrhea, abdominal cramps, greasy stools, bloating or gas, nausea, vomiting, weight loss, and dehydration (Figure 1). Approximately 50–75% of *Giardia*’s infections are asymptomatic [6]. Giardiasis is increasing significantly in developed countries, due to travel patterns and the globalization of society [7]. In fact, it is estimated that, for example, in the United States, more than 1 million people are infected with this parasite every year, with children being at higher risk than adults [8]. Untreated, it can have long-term complications, such as reactive arthritis, irritable bowel syndrome, and recurring diarrhea [2], and it can jeopardize a child’s development. Importantly, *G. intestinalis* resistance to metronidazole, a clinically important drug of choice against protozoa, has been reported [9]. Hence, the treatment of the infection caused by this pathogen is difficult, due to its effective pathogenesis, as well as the antibiotic resistance.

An effective vaccine would be an important tool to interrupt the transmission and prevent infections of this disease. The induction of the immune system via a vaccination using antigens obtained from the pathogens is a powerful strategy to produce antibodies, as well as a memory of pathogens. It has been accepted that vaccines do not only reduce the chance of infection, but they also help to mitigate disease severity in the case that a person becomes infected. Indeed, Lee et al. [10] reviewed on the importance of vaccination against *Giardia* about 10 years ago, but thus far, no effective human vaccine has been approved. A functional vaccine with total protein extract from a culturally cultivable sheep isolate (S2) has been developed and used in cats and dogs [11,12]. *Giardia* Vax^TM^ (Fort Dodge Laboratories, USA), a crude veterinary vaccine with a concoction of trophozoite lysates of sheep, dogs, and human isolates developed in 1999, has been approved for cats and dogs, but showed no indication of ever working in humans, especially upon conflicting data on their effectiveness in these animals [12,13]. In fact, its production has been stopped due to low efficacy, although ironically, it is still commercialized in some countries, such as USA, Brazil, Argentina, and Australia for use in dogs [14,15], even with new funding for anti-*Giardia* veterinary drug development [16]. Later, an antigen vaccine based on α1-giardin showed promise in murine models [17], but its validity in higher vertebrates is unknown, although research in vaccines with this antigen candidate continues [13]. Several current studies have examined the protective effect of subunit vaccines with several antigenic protein candidates [18] and peptide-based vaccines incorporating T-cell and B-cell epitopes [19].

Concomitantly, nanotechnology has gained prominence in a wide variety of applications, as it can be tuned to reach desirable attributes by handling physicochemical properties, such as size, shape, and charge [20]. Nanoparticles are tiny materials, whose size ranges from 1 to 100 nm and beyond, with varied applications in biology and medicine [21]. They could re-establish the use of poisonous drugs (such as metronidazole or quinacrine used to treat giardiasis) though the use of complex structures that allow for transporting drugs only into pathogens and preserving host cells, hence exerting their effect with less toxicity, improved selectivity, and therefore, greater efficiency. Moreover, nanomaterials have been used as antigen delivery vehicles and adjuvants to elicit prophylactic immune responses [22,23], namely against cancer, but also against other infections, such as the current COVID-19 pandemic [24,25]. The application of nanomaterials in *Giardia* vaccines is an exciting and recent development.

Therefore, this study aimed to describe the *G. intestinalis* antigenic proteins that served as candidate vaccines. Furthermore, we proposed micro- and nanoparticles and lipid-based delivery systems used as the innovation to transport antigenic molecules. Finally, the immune response in the presence of antigens was also described.

## 2. Methods

In this review, we collected the relevant findings from literature, such as original articles and reviews. A literature search was performed in September 2022 using PubMed for “*Giardia* vaccine”, “Giardiasis in human”, “*Giardia* antigens”, “*Giardia* candidate antigens”, “*Giardia* vaccine + nanotechnology”, “*Giardia* vaccine + drug delivery”, and “immune responses of *Giardia* infection”. For the complementary search, Google Scholar was used.

## 3. Antigenic Protein Candidates in *Giardia*

*G*. *intestinalis* can be divided into eight assemblies of A, B, C, D, E, F, G, and H, based on the genetically different housekeeping genes [26,27]. All assemblies have been found in the intestine of several vertebrate hosts. The assemblage A and B infect humans (WB and GS isolates, respectively) [28], cats, and dogs [29,30]. These are the two major genotypes of G. *intestinalis* that infect humans, but are genetically and biologically very different between themselves, so that they may warrant separate subspecies designations [5]. The GS/H7 isolate of *G*. *intestinalis* (assemblages B) has been reported to infect humans and mice [31]. Several *G*. *intestinalis* proteins are known to be present in immune sera in infected humans and animals [32]. They have been found on the surface of trophozoites in mouse models and may provide protection by vaccination (Table 1).

A family of variant-specific surface proteins (VSPs) are the best-characterized *Giardia* antigens recognized by hosts [33]. VSPs have a signal peptide (SP), a conserved C-terminal region with a single transmembrane domain (TMD), and a short cytoplasmic tail (CT) of just five amino acids (CRGKA) [34]. VSPs are a family of cysteine-rich proteins commonly found in *Giardia* trophozoites. *Giardia* can persist in harsh environments. It has been hypothesized to depend on the VSPs’ proteolytic digestion resistance, CXXC motif-provided characteristics, and metal-binding (iron and zinc) abilities [35]. Additionally, because VSPs are engaged in the process of antigenic variation, the parasite can change the expression of antigenically distinct VSPs, to evade the continual immunological pressure created by their hosts [36]. In addition to VSPs, there are other proteins classified in cysteine-rich proteins, such as Cys-rich membrane proteins (CRMPs) and secretory Cys-rich proteins (SCRPs). However, CRMPs and SCRPs are also encoded in the Giardia’s genome, but their functions are controversial [37]. Over 136 VSP genes have been identified in the Giardia genome [37], of which only one VSP is expressed on the cell surface at any given time; however, a switch in expression to an antigenically distinct VSP has been reported to occur spontaneously [31]. One of the two surface-resident VSPs is lost during switching after 12–36 h, and if the process is considered linear, the half-life is 17.3 h. The dynamics of VSP flipping resembles those of other parasites that undergo antigenic changes both topically and mechanically. It can take between 6.5 and 13 generations in culture before VSP switches, and this propensity is strain- and VSP-dependent. Therefore, a variety of expressed VSPs can be found in most cultures [38]. The genetic groupings of any trophozoite determine whether a certain VSP is present or absent from it. In other words, various *Giardia* genetic groups appear to have unique VSP repertoires that are more like each other than other genetic clusters. Using RFLPs, Nash et al. [39] divided *G*. *intestinalis* isolates into three groups in their initial relevant analysis. Group 1 and Group 2 were comparable to Group 3, but they were also noticeably distinct from it. A panel of molecular and phenotypic traits, including the existence of *vsp* genes and their capacity to express, was used to validate and broaden this classification [28]. The categories were later verified by other, more quantitative investigations, which aggregated the isolates from Groups 1 and 2 into Assemblage A and Group 3 into Assemblage B [5]. A well-known member of the *G*. *intestinalis* VSP family is VSPH7. Parasite surface antigenic variation is causally linked to VSPs. Both in vitro and in experimental human and animal infection-specific antibody responses to *G*. *intestinalis* and/or VSP infections have been shown to exhibit antigenic diversity [38]. The principal target of the humoral immune response in the neonatal mouse model using the *G*. *intestinalis* clone GS/M-83-H7 for experimental infection was found VSPH7 [40,41,42]. Rivero et al. [43] demonstrated that *Giardia* clones that express a particular VSP on their surface or cysts taken from infected individuals are mostly unable to infect the gerbil model, which was initially infected with cells that expressed all of the VSPs contained in their genome. These findings represent the experimental proof that antigenic diversity is crucial for parasite survival within hosts and that man-made interference with this mechanism may be valuable in developing vaccines against serious infections with similar behaviors.

In addition, several non-VSP antigens from *G*. *intestinalis* were identified, including cyst wall proteins (CWPs), α1- and α11-giardin, uridine phosphorylase-like protein-1, and protein 21.1. The encystation is a key process in the life cycle of *Giardia*, allowing the survival and transmission of this intestinal protozoan [44]. Increased pH and lack of available lipids in the distal ileum cause the encystment of *G*. *intestinalis* trophozoites to encyst. These conditions can be reproduced in vitro, where they cause the synthesis, trafficking, maturation, and deposition of three cyst wall proteins (CWPs 1–3), which are complexed with a special β-1,3-N-Acetylgalactosamine (GalNAc) glycan polymer, to start a differentiation process [45]. Genes encoding CWPs are entirely repressed in trophozoites and only expressed in encysting cells, in contrast to the CW glycan synthesis pathway, which is up-regulated during encystation [2,46,47]. The trophozoite binds to the intestinal microvilli during the vegetative stage of the parasite to colonize and withstand peristalsis. The concave structure of the cytoskeleton, known as the ventral disc, surrounded by a plasma membrane and with three different characteristics, enables the parasite to orient itself ventrally downward, towards biological or inert substrates (microtubules that coil around a bare area; microribbons that protrude into the cytoplasm; and cross-bridges that connect adjacent microtubules) [48]. rCWP2 are insoluble because of the protein-rich composition in cysteine residues and the hydrophobic leucine-rich repeat motif implicated in protein-protein interactions. These properties are an advantage for oral immunization, since they are able to resist to the harsh environment of the stomach, and therefore, its immunogenicity was preserved. Immunized mice shed fewer cysts after being exposed to live cyst challenges, which indicates that rCWP2 is a potential candidate antigen for the creation of a vaccine that prevents transmission [44]. Giardins are made up of three gene families, three of which are typically located in the ventral disc. These three gene families are I annexins (i.e., α-giardins), located on the outer edges of microribbons; (ii) bundles of striated fiber, such as β-giardin, which are closely associated with microtubules and δ-giardin (a component of microribbons); and (iii) g-giardin, which is also a microribbon protein [49]. A vast class of proteins, known as α-giardins, are encoded by 21 distinct genes (named a-1 to a-19). Although the structural protein α-2 giardin has been hypothesized to be a group A-specific proteins of human infective *G*. *intestinalis* [50], all 21 of these α-giardin genes in WB have been discovered to be conserved in GS, along with the genome synteny. Franzén et al. [51] found an α-2 giardin-like gene in the assemblage B GS strain, with 92% aa identity at the syntenic site, in a recent study. The discrepancies seen in crucial infection processes, such as adhesion and motility, between the two assemblages can be explained by variations in structural proteins. Since there is no mammalian counterpart of α-giardin, it is one of the most frequently detected conserved antigens by humans with giardiasis. This makes it a promising target for a vaccine. During human infections, antibodies against α-giardin begin to appear relatively early [52]. It is shed in the feces and expressed throughout the giardial life cycle, particularly in excysting cells that start an infection and trophozoites that cause sickness [53]. Among many *Giardia* isolates, α-giardin shows notable sequence conservation and immunological cross-reactivity, which is significant [17]. There is a possibility that the protection provided by immunization with α-giardin may be partially mediated by cross-reactivity with other giardin.

**Table 1:** Antigenic protein candidate vaccines and challenge studies performed in mouse models.

Protein	Antigen Template/Challenge Strain	Outcome	Model	Ref.
VSP H7	G. *intestinalis* clone GS/M-83-H7	Increased serum IgG, IgM	Mouse	[54]
VSPs1267 whole protein	Template, *G. intestinalis* WB strain ATCC 50803	Developed local (intestinal secretory IgA (S-IgA)) and systemic (serum IgG)	Mouse	[55]
CWP2 whole protein	Template, *G. intestinalis* WB strain ATCC 30957	Increased fecal IgA, increased serum IgG, cyst shedding reduction 80%	Mouse	[44]
CWP2 (M6-CWP2 fusion protein)	Template, *G. intestinalis* WB strain ATCC 30957	Increased fecal IgA, cyst shedding reduction 63%	Mouse	[56]
CWP2 (M6-CWP2 fusion protein)	Template, *G. intestinalis* WB strain ATCC 30957	Increased fecal IgA, increased serum IgG, cyst shedding reduction 70%	Mouse	[57]
CWP2 whole protein	Template, *G. intestinalis* WB strain ATCC 30957	Increased fecal IgA, increased serum IgG, cyst shedding reduction 60%	Mouse	[58]
α1-g whole protein	Template, *G. intestinalis* WB strain ATCC 50803 Challenge, G*. intestinalis* GSM strain ATCC 50581	Increased fecal IgA, increased serum IgG, reduced trophozoite load by 80–90%	Mouse	[17]
5G8 protein (fusion protein)	Template, *G. intestinalis* strain ATCC 50581	Increased serum IgG2b, increased agglutination of trophozoites >70–90%	Mouse	[59]
CWP2 aa 248–363 α1-g whole protein	Template, synthesized sequence based on GL50803_5435 (WB) Challenge, *G. intestinalis* C2 Template, *G. intestinalis* C2 Challenge, *G. intestinalis* C2	Increased fecal IgA, increased serum IgG, cyst shedding reduction 93%, reduced trophozoite load by 79%	Mouse	[60]

## 4. Micro- and Nanoparticles and Lipid-Based Delivery Systems

*Giardia* lives in the intestinal tract of most classes of vertebrates, including humans and other mammals [5]. Humoral immunity is considered necessary for the removal of *Giardia* trophozoites from the host intestine [11]. A successful oral vaccine should result in the activation of intestinal dendritic cells that produce high level of T helper (Th) cells and an increase in IgA- and IgG-bearing plasma cells has been associated with natural elimination of the parasite [11,61]. Due to the harsh environment in the gastrointestinal tract, the oral vaccine needs to survive in the low pH and degradation by digestive enzymes. Delivery system strategies have been developed to protect and preserve the structural integrity of antigens and promote vaccine penetration and enable their release within the induction of immunity. Delivery systems in non-living systems, including virus-like particles (VLPs), micro-/nanoparticles (NPs), and nanogels, have also been developed [61].

Polymeric micro-/nanoparticles and lipid-based vehicles have been used to deliver vaccine antigens to the induction sites. Three advantages of this strategy are (i) the encapsulation of antigens in particles can prevent antigen breakdown and improve antigen persistence of antigens. (ii) Antigen-presenting cells (CPAs), such as macrophages and dendritic cells, have been shown to easily phagocyte particles ranging in size from 150 nm to 4.5 µm [62,63]. (iii) More particle-based platforms may be designed to contain additional adjuvants and/or targeting moieties to further influence immunogenicity [64,65]. Several particle-based antigen delivery techniques, including liposomes, immune-stimulating complexes (ISCOMs), and polymeric particles, are under development and have been reviewed in the pathogens listed in Table 2. It has been reported that high titers of IgG are produced after vaccination with a liposomal formulation of the NKT cell antigen PBS57 and the oligosaccharide epitope in PBS150 [66]. In addition, interbilayer crosslinked multilamellar vesicles lipid nanoparticle vectors enhanced a range of quantitative and qualitative features of the immune response to the recombinant protein antigen derived *Plasmodium vivax* [67]. Among the nanoparticles for vaccine delivery, only polymeric nanoparticle has been used with encapsulated *Giardia* membranes. Nano-vaccines have been developed against *G. intestinalis* by coating membranes derived from *G. intestinalis* WB ATCC 50803 and GS/M ATCC 50581 in homogeneous and consistent polymeric nanoparticles loaded with the mucosal adjuvant cholera toxin (CTX). Intranasal immunization with the nano-vaccine induced adaptive immunity and effectively protected mice from *G. intestinalis* infection [68].

## 5. Immune Responses against *Giardia*

### 5.1. Innate Immune Responses to Giardia

Innate immunity protects the host by the infection of the pathogen via many mechanisms, such as the first line of defense in the immune response, humoral substances, and the immune’s cells without the specificity. Antimicrobial peptides (AMPs) are the innate immune system in the small intestine, which are produced by Paneth cells and secreted into the intestinal lumen for the maintenance of the mucosal barrier [77]. AMPs have previously been reported to inhibit *Giardia* trophozoites in vitro. Defensins and indolicidin are antimicrobial polypeptides that may play a role in reducing the viability of *G*. *intestinalis* by more than 3 log units in 2 h. The dose and time dependency of all peptides were observed for trophozoites killing. The morphology of trophozoites was changed after exposure to peptides [78]. Lactoferrin and its N-terminal peptides had anti-*Giardia* activity in vitro. Trophozoites in log-phase cells were much more resistant than stationary-phase cells to killing by lactoferrin and its N-terminal peptides [79]. Nitric oxide (NO) is produced by intestinal epithelial cells [80]. It is a broad-spectrum antimicrobial against many bacterial and parasitic pathogens [81] and has a functional epithelial barrier [82]. NO possesses anti-*Giardia* activity, in terms of cytostatic and exhibited inhibition encystation and excystation for *G*. *intestinalis* [80]. However, in models of the human intestinal epithelium, *G*. *intestinalis lamblia* has strategies to evade this potential host defense by consuming arginine affecting the inhibition of NO production [80]. Upon mice model infections, identified macrophages containing digested *G*. *muris* were surrounded by rosettes of lymphoblasts in the epithelium [83], and in vitro studies showed that human monocytes and macrophages have the potential to ingest *Giardia* trophozoites, which are subsequently killed by an oxidative mechanism [84]. Mast cells have been used to study helminth infection models, where they associate with parasite elimination, depending on the parasite species [85]. Previous studies reported that mast cells were alternative antigen-presenting cells that might play an important role in controlling infections with both *G*. *muris* and *G*. *intestinalis* [86,87]. Mast cells are recruited to the intestine, where they degranulate. In addition to releasing histamines and mouse mast cell protease-1 (MMCP-1), previous work has shown that they may interact with cholecystokinin (CCK), which, in turn, leads to increased intestinal contractility. Treating tissues with compounds inhibiting mast cell degranulation (ketotifen) or depleting them of granule contents (compound 48/80) removes the effects of CCK [87].

### 5.2. Adaptive Immune Response to Giardia

The adaptive immunity is produced by the exposure of various antigens to the immune cells, resulting in the production of a specific antibody, as well as the memory to those of the antigens. In the last decade of studies of *Giardia* infections of animals, interleukin 17 (IL-17) has been discovered to have an essential role in the control of these infections [88]. IL-17 is an important component of broad immunity to candidiasis, caused by *Candida albicans*, and promotes the expression by neutrophil and epithelial cells [89]. Solaymani-Mohammadi and Singer [90] provided the first report of the synthesis of IL-17 in mouse spleen cells and MLNs after *G. intestinalis* infection. Like in *G. intestinalis* infection, IL-17 production was first reported in the mouse response to *G. muris* after three weeks post-infection. The lack of IL-17A receptors in mice showed a high level of cyst release in feces, compared with wild-type mice. This report exhibited that IL-17A was important for elimination of *G. muris* infections [91]. IL-17 production was stimulated by several immune cells during protozoan infection. In mice, infection with *G. muris* and *G. intestinalis* indicated an upregulation of CD4^+^ T cells producing IL-17 in the lamina propria and innate immune cells in the epithelial compartment of the small intestine [92]. Likewise, innate lymphoid cells (ILCs) group 3 from the lamina propria secretes IL-17 in response to *G. intestinalis* [93]. In human giardiasis, the IL-17A revealed an important protective immune response against *Giardia*. The study was a case of individuals in Norway who were exposed to *Giardia* while traveling abroad. The researchers used flow cytometry to detect the immune response to *Giardia* infection, and they found an upregulation of IL-17A and TNF production by CD4^+^ CD197^−^ CD45RA^−^ T cells [94]. These data demonstrated that IL-17 has a crucial role in human giardiasis, as well as in mouse models. Immunoglobulin A (IgA) is an important antibody in the intestinal mucosa for controlling *Giardia* infection. Previous studies have reported that *Giardia* infection induces a robust IgA response. Secretory IgA antibodies (sIgA) levels were significantly higher in the serum or salivary of *Giardia*-infected individuals, compared with other protozoa infections and the non-parasitized group [95]. Studies in mice models found that IgA levels in serum were stimulated by oral administration excretory/secretory products (ESP) derived from *Giardia* and soluble *Giardia* extract [96]. Variant-specific surface proteins, or VSPs (VSP3 and VSP5), are the targets of anti-*Giardia* IgA antibodies. IgA levels in patients’ sera against VSP3 and VSP5 were followed by flow cytometry immunoassay. Hjøllo et al. [97] found that the levels of anti-VSP5 and anti-VSP3 IgA decreased after treatment but were still higher than those of the uninfected control group. Moreover, they presumed that the rapid fall in IgA levels after treatment could induce reinfection in individuals.

## 6. Conclusions

The global disease burden from infectious illnesses is still very high. Everyone agrees that the best defense against infectious diseases is vaccination. Moreover, the use of *Giardia* vaccines in animals could have implications for reducing the transmission of infections in animals to susceptible hosts. These consistent findings of vaccines in different animal species make the creation of the *Giardia* vaccine for humans possible. However, prior to the creation and testing of *Giardia* vaccines in production animals, several technical questions must yet be resolved. This review shows that the discovery of conserved surface antigens offers a potent strategy for circumventing a crucial rate-limiting step in the development of a potent giardiasis vaccine. The future of nanotechnology in the delivery system is promising. It would result in a simultaneous improvement in the quality, efficacy, and safety profile of the vaccine.

## Figures and Tables

**Table 2:** Types of nanoparticles studied for antigen delivery in parasites and other microorganisms.

Type of Drug Delivery System	Nanoparticle Material	Size	Antigen (Pathogen)	Ref.
Inorganic (non-degradable)	Iron silica	20–300 nm	MSP1 (*Plasmodium falciparum*) BSA	[69] [70]
Liposome (non-viral lipids particle)	Cholesterol lipid lipid	200 nm	Polysaccharides (*Streptococcus pneumoniae* serotype 14) VMP001 (*Plasmodium vivax*) RTS,S/AS01B (*Plasmodium falciparum* CSP + hepatitis B protein hybrid)	[66] [67] [71]
Polymeric	Chitosan PLGA PLGA PVPON_Alk_ γ-PGA PLGA	160–1000 nm	Hepatitis B Ovalbumin Tetanus toxoid Ovalbumin gp120 (HIV-1) Membrane vesicles (*G. intestinalis* WB ATCC 50803 and *G. intestinalis* GS/M ATCC 50581)	[72] [73] [74] [75] [76] [68]

## Data Availability

Not applicable.
